# Current and Promising Approaches to Identify Horizontal Gene Transfer Events in Metagenomes

**DOI:** 10.1093/gbe/evz184

**Published:** 2019-08-26

**Authors:** Gavin M Douglas, Morgan G I Langille

**Affiliations:** Department of Microbiology and Immunology, Dalhousie University, Halifax, Nova Scotia, Canada

**Keywords:** horizontal gene transfer, lateral gene transfer, shotgun metagenomics, metagenome-assembled genomes, microbiome

## Abstract

High-throughput shotgun metagenomics sequencing has enabled the profiling of myriad natural communities. These data are commonly used to identify gene families and pathways that were potentially gained or lost in an environment and which may be involved in microbial adaptation. Despite the widespread interest in these events, there are no established best practices for identifying gene gain and loss in metagenomics data. Horizontal gene transfer (HGT) represents several mechanisms of gene gain that are especially of interest in clinical microbiology due to the rapid spread of antibiotic resistance genes in natural communities. Several additional mechanisms of gene gain and loss, including gene duplication, gene loss-of-function events, and de novo gene birth are also important to consider in the context of metagenomes but have been less studied. This review is largely focused on detecting HGT in prokaryotic metagenomes, but methods for detecting these other mechanisms are first discussed. For this article to be self-contained, we provide a general background on HGT and the different possible signatures of this process. Lastly, we discuss how improved assembly of genomes from metagenomes would be the most straight-forward approach for improving the inference of gene gain and loss events. Several recent technological advances could help improve metagenome assemblies: long-read sequencing, determining the physical proximity of contigs, optical mapping of short sequences along chromosomes, and single-cell metagenomics. The benefits and limitations of these advances are discussed and open questions in this area are highlighted.

## Introduction

Microbes are microscopic organisms that include prokaryotes (bacteria and archaea), viruses, fungi, and protists. These organisms, particularly prokaryotes and viruses, are known to rapidly adapt to novel abiotic and biotic environmental changes. The genetic bases for these adaptations have largely been identified by studying the genomes of isolated organisms of interest, which have greatly improved our understanding of the genetic bases of adaptations throughout microbial evolution (Parkhill et al. 2003; Etienne et al. 2013; Harding et al. 2017).

A substantial proportion of microbes in natural communities are currently uncultured (Amann et al. 1995; Martiny 2019) and acquiring isolate genomes for these organisms has proven difficult. These uncultured microbes have been studied through metagenomics approaches, which involve the sequencing of all, or an enriched set, of microbial genomes in a sample (Riesenfeld et al. 2004). Metagenomics sequencing (MGS) enables researchers to investigate how environmental conditions shape the taxonomic and functional composition of natural microbial communities. Recently, shotgun MGS—unbiased high-throughput sequencing of all DNA in a sample—has become the predominant method of metagenomics profiling. MGS analyses have largely been gene-centric, meaning that the focus has been on the relative abundances of individual genes (and inferred pathway relative abundances) in a community. More recently, the focus has shifted toward generating metagenome-assembled genomes (MAGs) from this sequencing data (Parks et al. 2017; Stewart et al. 2018; Pasolli et al. 2019). An important challenge for either analysis approach is to determine whether genes hypothesized to be adaptive arose through gene gain mechanisms or alternatively were part of preexisting genetic variation.

New genes can be acquired through three processes: 1) gene duplication and diversification, 2) the gain of a de novo gene (e.g., in previously noncoding DNA), and 3) horizontal gene transfer (HGT). In addition to the gain of new genes, microbes are known to adapt to new environments through gene loss. All of these processes will be described in detail below.

Although most MGS analyses are based on gene-centric methods, assembling MGS reads into genomes is one clear way to improve the identification of gene gain and loss events. The key challenge of this approach is that assembly errors can result in gene gain and loss events being falsely identified or missed. Several recent technological advancements could help overcome this barrier. These technologies include long-read sequencing, mapping the physical proximity and interactions of DNA fragments, optical mapping of short sequences along chromosomes, and single-cell metagenomics.

**Fig. 1.**
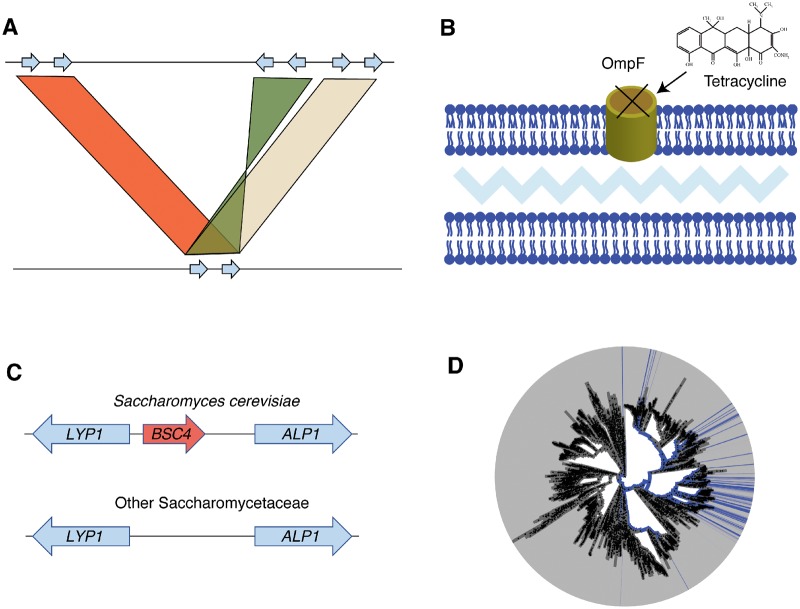
**—**Examples of microbial gene gain and loss. (*A*) Illustration of operon duplications between two genomes. Arrows indicate genes and colored bars indicate different regions of homologous DNA shared between the two genomes. This simplified example is inspired by the mercury resistance operon duplications identified in *Rhodanobacter* genomes (Hemme et al. 2016). High levels of mercury resistance genes were reported in groundwater metagenomes dominated by *Rhodanobacter*, but genomic analyses were required to identify putative duplication events. (*B*) An example of adaptation through loss-of-function. Tetracycline (indicated by chemical structure) is largely imported through the OmpF porin in *Escherichia coli*. Deleting the gene encoding this porin allows for higher tetracycline tolerance (Thanassi et al. 1995). (*C*) Example of de novo gain of the *BSC4* gene in *Saccharomyces cerevisiae* compared with other Saccharomycetaceae (Cai et al. 2008). Simplified visualization of orthologous region across fungi demonstrates that *BSC4* is only present in *S. cerevisiae*. (*D*) Distribution of two KEGG orthologs (Kanehisa and Goto 2000) (K08928, K08929), which are responsible for anoxygenic photosystem II (M00597), that are broadly distributed across the prokaryotic tree likely due to horizontal gene transfer. The presence and absence of these gene families is indicated in blue and gray, respectively. Panel created with AnnoTree (Mendler et al. 2019).

Herein, the approaches applied so far to detect gene gain and loss events in MGS data are reviewed. This review largely focuses on identifying HGT events because these events are of primary interest within the field and there have been several recent methodological advances in HGT-detection in the context of metagenomes. However, methods to identify alternative processes of gene gain and loss will first be discussed (fig. 1). The background and mechanisms of HGT are then described, followed by a comparison of the tools applied to detect HGT within assembled genomes and MGS data. Lastly, several recent technologies are described that could help address the issue of producing high-quality MAGs from MGS data.

## Gene Gain and Loss Events

Although HGT is the predominant mode of gene gain studied in MGS data, several other mechanisms of genic gain and loss are important to consider: gene duplication, gene loss, and de novo gene birth (fig. 1). The relative importance of these processes, and HGT, in driving adaptive evolution in microbes remains unclear. However, profiling the occurrence and adaptive benefit of these events could help resolve this issue. These genic events will be described and possible methods for identifying them in MGS data will be discussed below.

### Gene Duplication

Gene duplication has long been known to be an important process underlying adaptation to novel environments (Kondrashov 2012). In prokaryotes, gene duplication events are typically at the order of individual genes and operons, particularly of genes involved with transcription, metabolism, and defense (Gevers et al. 2004). Duplication of individual genes can similarly occur in eukaryotic genomes, but eukaryotes also commonly undergo larger genomic duplication events, such as whole-genome duplications (Aury et al. 2006). The vast majority of duplicate genes gain degenerative mutations and become nonfunctional pseudogenes (Lynch and Conery 2000); however, surviving genes can acquire divergent or novel functions (Ohno 1970; Force et al. 1999). Regardless of the mechanism, gene duplicates are extremely common in nature: an estimated 7–41% of bacterial proteins are encoded by paralogs (Gevers et al. 2004). Duplicates can also be retained without diverging in function as well, which can provide higher protein expression levels (Schuster-Böckler et al. 2010; Kondrashov 2012) or can keep dosage levels proportional to other gene duplicates (Conant et al. 2014).

Mapping unassembled reads to gene family databases can be used to generate hypotheses about gene duplications, which can be evaluated with additional data. For example, high abundances of mercury resistance genes were observed in groundwater metagenomes dominated by *Rhodanobacter* (Hemme et al. 2016) and subsequent analyses identified putative duplicated mercury resistance operons within *Rhodanobacter* genomes (fig. 1*A*). However, in general, gene duplicates are difficult to identify in MGS data by characterizing the relative abundances of gene families within metagenomes. This challenge is largely due to the difficulty of distinguishing paralogous sequences from the same genome from orthologs across multiple genomes. In addition, most methods for detecting gene duplicates, and structural variants (SVs) in general, are intended to be applied to genome resequencing data of a single organism mapped against a reference genome (Ye et al. 2009; Rausch et al. 2012; Yavaş et al. 2014). Applying such approaches to identify SVs in a mixed community would likely result in widespread false inferences. The exceptions could be when a community is dominated by a small number of known species or if a subset of reads can be confidently binned into species. In these cases, mapping species-identified MGS reads to the appropriate reference genomes and then applying existing SV-detection methods would be reasonable. Although these methods are optimized for the human genome, previous work has shown that prokaryotic strain-level SVs can be accurately identified using a consensus of multiple tool outputs (Zojer et al. 2017).

No single pipeline is available for identifying gene duplicates in fragmented metagenomes, but numerous approaches could be leveraged by comparing MAGs with existing reference genomes. For instance, comparative genomics could be employed to identify clusters of homologous genes across genomes using reciprocal BLAST matching between assembled contigs and reference genomes of closely related taxa (*Drosophila* 12 Genomes Consortium et al. 2007; Hahn et al. 2007). Under this approach, orthologous genes are typically assumed to be reciprocal best-hits whereas other similar genes are putative paralogs (Ward and Moreno-Hagelsieb 2014). There are several methods available for summarizing species pan-genomes as well, such as Roary (Page et al. 2015) and panX (Ding et al. 2018), which could be useful for interpreting gene duplication patterns across genomes. In addition, although putative gene duplicates could be identified through comparison with known reference genomes it is possible that alternative explanations such as gene loss or HGT could better account for this signature, which would need to be reconciled (see the Identifying HGT Events in Metagenomes section). It is also important to emphasize that misassembled MAGs might be especially susceptible to false inferences of gene duplication, especially if multiple closely related taxa are included in the same assembly.

### Gene Loss

Gene loss is normally associated with decreased negative selection, but recently the importance of gene loss in adaptive evolution has been demonstrated (Hottes et al. 2013; Albalat and Cañestro 2016). Many adaptive loss-of-function (LOF) mutations knockout an individual protein, such as the deletion of the porin *ompF* locus in *Escherichia coli*, which grants tolerance to tetracycline by preventing its import (Thanassi et al. 1995) (fig. 1*B*). However, there are also cases of adaptive LOF mutations disrupting regulatory networks, such as the knockout of genes in *Candida glabrata* required to synthesize nicotinic acid, which causes epithelial adhesion genes to be expressed and enables binding to murine renal cells (Domergue 2005). For segregating knockout mutations, there are existing tools for profiling strain-level variation, including single-nucleotide polymorphisms, relative to reference genomes (Nayfach et al. 2015; Scholz et al. 2016; Costea et al. 2017). The output of the identified mutations could be used with existing programs such as SnpEff (Cingolani et al. 2012), which predict the effect of mutations on protein-coding genes. One potential issue with this approach is that misaligned reads could result in many false LOF mutations being identified. Focusing analyses instead on MAGs could be a more straight-forward way to identify LOF mutations. However, these LOF mutations could result in genes not being annotated in assembled contigs if they are not identified to be open-reading frames. Also, large deletions would result in genes missing entirely from MAGs. In this case, gene loss would need to be inferred by identifying orthologous regions of DNA using similar approaches as described above for identifying gene duplicates.

Alternatively, the absence of genes could be identified if they are annotated in closely related reference genomes. For example, the ribosomal proteins L9 and L1 were recently shown to be differentially absent within multiple MAGs from the poorly characterized candidate phyla radiation group (Brown et al. 2015). These ribosomal proteins are otherwise highly conserved in bacteria, so this finding indicates a shift in ribosome function within this lineage. These instances of gene loss were identified consistently across multiple MAGs, which provides additional evidence that this finding is not a technical artifact.

### De Novo Gene Birth

De novo gene birth is caused by mutations that give rise to novel protein-coding regions, which fall into two categories. The first category is de novo genes arising in preexisting protein-coding regions, typically on the opposite strand or a separate reading frame, which is referred to as overprinting. This form of de novo gene birth was first described in the genome of the virus phiX174 (Weisbeek et al. 1977) and numerous other cases have since been identified in viruses (Sabath et al. 2012; Van Oss and Carvunis 2019) and bacteria (Ohno 1984; Hücker et al. 2018; Vanderhaeghen et al. 2018). One example is of the transcript *nog1* found on the reverse strand of the gene *citC* in *E. coli* (Fellner et al. 2015). Although the function of this gene remains unclear, it was shown that this gene likely encodes a protein, based on experimental evidence and the presence of a Shine–Dalgarno sequence upstream of the predicted start codon. In addition, knocking out *nog1* while maintaining the citC amino acid sequence resulted in decreased fitness relative to wild-type. Identifying such cases of overprinting is likely not feasible within MGS data sets alone and instead meta-transcriptomics data would be required to identify alternative transcripts originating from the same locus. One major challenge facing this approach would be distinguishing transcripts encoding proteins from antisense transcripts producing noncoding RNAs involved in gene regulation (Brantl 2007; Bao et al. 2015).

The second type of de novo gene birth occurs when protein-coding regions arise from noncoding DNA (Tautz and Domazet-Lošo 2011). This form of de novo gene birth is known to occur in eukaryotes, especially at low frequencies in a population, but these cases are largely nonadaptive and undergo pseudogenization (Tautz and Domazet-Lošo 2011). Nonetheless, there are many examples of such de novo genes conferring adaptive benefits (McLysaght and Guerzoni 2015; Schlötterer 2015). One example is of the *BSC4* gene in *Saccharomyces cerevisiae*, which is unique to that species (Cai et al. 2008) (fig. 1*C*). This gene encodes a protein involved in the DNA repair pathway during stationary phase (Cai et al. 2008). Importantly, such examples of de novo gene birth are restricted to eukaryotes, likely due to the low proportion of noncoding DNA in prokaryotic genomes. Existing methods (Van Oss and Carvunis 2019) could be leveraged to identify these cases within high-quality eukaryotic MAGs. One possible approach would be to identify candidate de novo genes in a MAG that are homologous with noncoding DNA in all related taxa.

## Signatures of HGT

HGT, also known as lateral gene transfer, is the transfer of genetic material outside parent–offspring inheritance. Importantly, HGT differs from the other processes discussed above because it enables the rapid transfer of genes between distantly related species and is thought to be especially important for the adaptation of microbes to novel environments (Ochman et al. 2000). In particular, there is a heightened interest in HGT due to concerns regarding the spread of antibiotic resistance genes in hospitals (Spellberg et al. 2008), livestock (Mathew et al. 2007), and waterways (Szczepanowski et al. 2009; Bengtsson-Palme et al. 2014). More generally, the importance of HGT for microbe niche specialization is demonstrated simply by the sparse distribution of key functions across life (Boucher et al. 2003). One representative example is of tetrapyrrole-based photosynthesis, which is patchily distributed across bacteria likely due to HGT (fig. 1*D*).

There are numerous mechanisms underlying HGT, which differ in frequency across taxa and depend on the genetic distance between the donor and acceptor genomes (Thomas and Nielsen 2005). The three main mechanisms are transformation, conjugation, and transduction. An understanding of these different mechanisms is important because they can be associated with distinct genomic signatures (e.g., specific sequences linked to a transfer mechanism), which can be corroborating evidence of HGT (reviewed in Zaneveld et al. [2008]).

Transformation refers to the uptake and integration of extracellular DNA and commonly occurs across prokaryotes. DNA uptake requires a cell to be in a physiological state known as competency, which typically involves the activity of 20–50 proteins (Thomas and Nielsen 2005). The transformation rate in an environment depends on both the proportion of competent cells and on the concentration of extracellular DNA, which greatly varies across environments. For instance, concentrations of extracellular DNA in marine sediments are typically three orders of magnitude higher than in marine water (Torti et al. 2015). Input DNA becomes single-stranded when translocated across the inner membrane and the translocated DNA can then undergo homologous recombination with similar sequences or be used as a source of nutrients (Finkel and Kolter 2001). Theoretically, the potential for these transfer events might be inferred by identifying the presence of genes encoding proteins involved in this process (Zaneveld et al. 2008). For instance, there are many surface proteins involved in the uptake of extracellular DNA, some of which are similar to surface appendages such as type IV pili (Chen and Dubnau 2004). These proteins are well characterized in a small number of bacteria such as *Bacillus subtilis* and *Streptococcus pneumoniae* (Claverys et al. 2009) and could be used as markers for the potential to undergo transformation in these species. However, scanning for the genes encoding these proteins in the genomes of poorly characterized species would likely not yield accurate identification of the potential for DNA uptake.

Conjugation is a mode of unidirectional DNA transfer through cell-to-cell junctions, typically mediated by type IV secretion systems that only transfer single-stranded DNA (Zaneveld et al. 2008). Conjugation is the predominant mechanism of transfer of plasmids (Thomas and Nielsen 2005) (which are transferred as single-stranded and circularized copies) and is especially relevant for the spread of antibiotic resistance (Akiba et al. 1960; Kristiansson et al. 2011; Bengtsson-Palme et al. 2014). Many conjugative plasmids are self-transmissible and can either integrate into the host genome or remain autonomous in the cell (Yin and Stotzky 1997). Conjugal transfer is typically between closely related organisms but can also occur between distantly related taxa (Dahlberg et al. 1998; Lacroix and Citovsky 2016). The most well-known proteins involved in forming the cell–cell junction are the *tra* proteins, which are conserved in certain lineages (Lanka and Wilkins 1995). The origin of transfer (*oriT*) is an element within plasmid DNA that specifies where the relaxase protein binds (Grohmann et al. 2003). Relaxase binding promotes the formation of the relaxosome complex, which nicks the DNA at a conserved DNA motif called *nic* (Zaneveld et al. 2008). Identifying the presence of these proteins and DNA motifs in metagenomics data could be used to infer the potential for conjugation. However, the most straight-forward signature for potential conjugation in MGS data is the presence of plasmids.

There are several approaches that have been developed for identifying plasmid sequences in MGS data. One approach is to identify circular contigs in assembly graphs, as performed by the software Recycler (Rozov et al. 2017). Another major approach is to compare reads or contigs to a database of reference plasmids (Carattoli et al. 2014), which restricts researchers to previously identified plasmids. Lastly, differing k-mer profiles between chromosomal and plasmid DNA can be leveraged using machine learning approaches to identify novel plasmid sequences (Zhou and Xu 2010; Krawczyk et al. 2018). An especially promising tool using this approach is PlasFlow, which classifies chromosomal and plasmid contigs with a neural network trained on reference sequence k-mer content (Krawczyk et al. 2018). This tool performed substantially better than the other approaches described above and provides the added benefit of identifying linear plasmids, which is not possible with assembly-based plasmid identification tools. These approaches for identifying plasmid sequences in MGS could be used to both establish that transferrable plasmids are present in the community and to identify genes contained on plasmids that could potentially be transferred.

The final major mechanism of HGT is transduction, which is the form of genetic transfer mediated through phage and can be categorized as generalized or specialized transduction (Yin and Stotzky 1997). Generalized transduction refers to the packaging of random DNA fragments from a bacterial genome into a phage capsid. This process can occur when a host cell is infected by either a virulent phage or a temperate phage undergoing a lytic cycle. In contrast, genes transferred through specialized transduction are integrated into the genomes of temperate phages when they are incorrectly excised from the host genome. Specialized transduction cannot involve virulent phages because integrating into the host genome as a prophage is a required step. Transferred genes are integrated along with the phage genome into new hosts (Yin and Stotzky 1997). Bacteriophage tropism is generally restricted to hosts within a single species (Koskella and Meaden 2013), although there are exceptions (Malki et al. 2015), which means that overall transduction is less common between distantly related organisms. It is also important to recognize that acquiring novel genes can enable phages to adapt to novel niches (Hatfull and Hendrix 2011). In addition, the intermediate stage of transduction within phages enables genes to rapidly evolve, which can result in novel beneficial functions if they are eventually acquired by a bacterial host (Comeau and Krisch 2005).

The genomic signatures of transduction have the most potential to be used to identify past transfer events. These signatures can be identified for specialized transduction, but not easily for generalized transduction because DNA transfer through the latter typically occurs through homologous recombination of randomly packaged bacterial DNA. In contrast, phages capable of specialized transduction typically integrate at a specific *attB* site within host genomes through recombination with an *attP* element in the phage genome (Canchaya et al. 2003; Zaneveld et al. 2008). In addition, only genes located nearby prophages in a bacterial genome will be transferred through specialized transduction. It has previously been established that genes transferred by specialized transduction can be identified by whether they are nearby phage-related sequences, such as phage integrases, or are nearby transfer RNAs (Williams 2002), which is a preferential integration site for certain temperate phages. Accordingly, the presence of prophage sequences nearby putatively transferred genes could be taken as corroborating evidence of specialized transduction.

Approaches to identify prophage sequences in isolate genomes typically rely on sequence similarity with known viral genes as well as identifying regions with viral genome characteristics (Akhter et al. 2012). These characteristics include shorter protein lengths, shared transcription directionality of adjacent genes, and distinct k-mer profiles. Identifying viral sequences in MGS data is more complicated than in isolate genomes because the datatype is more diverse and fragmented, but nonetheless several tools have been developed for this purpose (Ren et al. 2017; Amgarten et al. 2018; Garretto et al. 2019). These tools have mainly been focused on identifying viral contigs in MGS data rather than large bacterial contigs containing a prophage, but there are exceptions. VirSorter is one approach that can explicitly identify prophages in MGS data (Roux et al. 2015). This tool scans contigs for the presence of viral genes and certain virus-like characteristics and reports the confidence that each gene, as well as the contig overall, is virus derived. PHASTER is a similar tool that identifies prophage regions in contigs based on the sequence similarity of open-reading frames with reference phage and prophage genes (Arndt et al. 2016). The output of these tools could be particularly useful for finding corroborating evidence of specialized transduction transfer events.

Although the three mechanisms discussed above are the best-studied modes of HGT, there are several other notable mechanisms. The foremost of these other mechanisms are gene transfer agents (GTAs), which are phage-like particles that transfer DNA between cells. At least five families of GTAs have been identified in prokaryotes (Lang et al. 2017). Although most of these GTAs have been identified in only a single species, homologs for the GTA family characterized in *Rhodobacter capsulatus* have been identified in numerous *Alphaproteobacteria* species (Lang and Beatty 2007). It remains unclear how widespread GTAs are in nature, but it has been suggested that they could represent a substantial proportion of diversity identified in the marine virome (Kristensen et al. 2010). Two additional mechanisms of genetic transfer related to conjugation are intercellular nanotubes (Dubey and Ben-Yehuda 2011) and membrane vesicles (Domingues and Nielsen 2017), which are conducted through direct cell–cell physical interactions and the release and uptake of extracellular vesicles, respectively. The importance of these mechanisms for HGT across prokaryotes also remains controversial (Ficht 2011; Grüll et al. 2018).

## Identifying HGT Events in Metagenomes

**Fig. 2.**
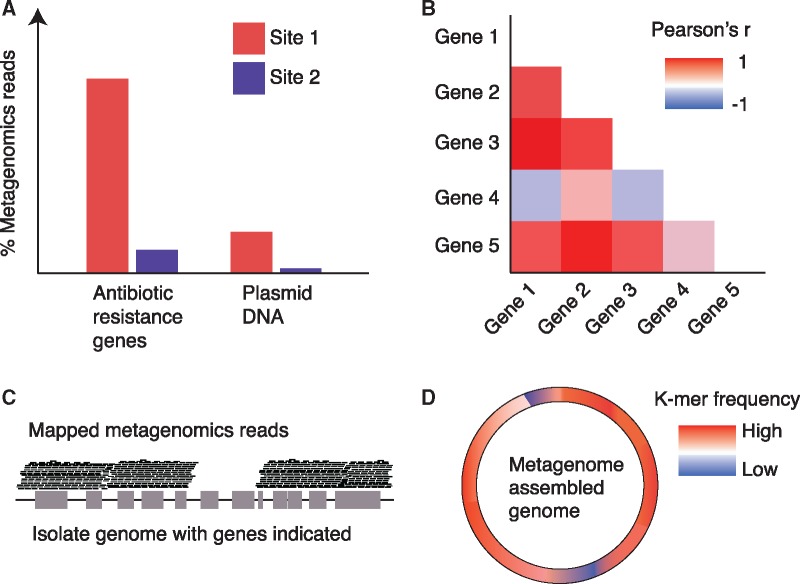
—Key approaches to infer potential HGT in MGS data. (*A*) Identifying genes frequently transferred through HGT at differential relative abundance between two sites. One possible explanation for these observations is HGT, which is frequently hypothesized in the literature although this is not based on direct evidence. (*B*) Identifying outlier genes in short assembled contigs using a compositional approach (Tamames and Moya 2008). This approach involves tabulating k-mer frequencies within each gene and calculating the pairwise Pearson correlation between all genes within the contig. Outlier genes with atypical k-mer composition can then be identified, which are candidates for HGT (such as gene 4 in this example). (*C*) Isolate reference genomes have been used with MGS data on several occasions. One example usage is to map the metagenomics reads to existing reference genomes to identify genomic regions not found in the metagenome. HGT is one possible explanation for the absence of reads mapped to a particular region of the reference genome as shown in this example. (*D*) Generating high-quality MAGs allows any method for identifying HGT in isolate genomes to be applied. The example shown here is of detecting genomic regions that have divergent k-mer composition compared with the rest of the genome. Note that in this simplified example only one k-mer is being compared whereas typically the profile of many k-mers would be compared.

Numerous approaches have been developed for detecting HGT in genomics data (table 1), which are largely intended to be applied to high-quality isolate genomes (Ravenhall et al. 2015). In contrast, although HGT is often hypothesized based on unassembled metagenomes, directly identifying putative HGT events is less common for this datatype (fig. 2*A*). For example, the higher relative abundances of antibiotic resistance genes and mobile elements, such as plasmids, in waterways downstream of wastewater treatment plants have been taken as putative evidence of the spread of antibiotic resistance through HGT (Szczepanowski et al. 2009; Bengtsson-Palme et al. 2014). Another example is that HGT has been suggested to be potentially responsible for higher similarity in chromium resistance genes than in 16S ribosomal RNA gene sequences found in water samples with high concentrations of chromium (Parnell et al. 2010). These and other similar studies (Kurokawa et al. 2007; Rua et al. 2018) represent the most common approach of inferring putative HGT in metagenomes. This approach can be useful for generating hypotheses but does not provide direct evidence for specific HGT events.

**Table 1:** Approaches for Identifying Putative Cases of HGT in Metagenomes

Approach	MGS Specific^a^	Example Implementations
Identify outlier genomic regions based on DNA composition	No	GIST (Hasan et al. 2012); IslandViewer (Bertelli et al. 2017)
Identify outlier genes in contigs based on pairwise Pearson correlations of k-mer content	Yes	Described in Tamames and Moya (2008)
Identify genes in genomes displaying taxonomically discordant similarity to genes within a reference database	No	DarkHorse (Podell and Gaasterland 2007); HGTector (Zhu et al. 2014)
Identify genes in contigs displaying taxonomically discordant similarity to genes within a reference database	Yes	WAAFLE^b^ and a method described in Tamames and Moya (2008)
Reconcile phylogenetic incongruencies between gene and species trees	No	AnGST (David and Alm 2011); RANGER-DTL (Bansal et al. 2018)
Identify putative donor and recipient transfer events within a given community based on a combined similarity and phylogenetic incongruency approach	Yes	MetaCHIP (Song et al. 2019)
Identify genomic hotspots of recombination between strains of a species	Yes	Described in Tyson et al. (2004) and Probst and Banfield (2018)

a Whether an approach is intended specifically for shotgun metagenomics (MGS) data instead of isolate genomes.

b http://huttenhower.sph.harvard.edu/waafle.

In contrast, identifying potential HGT events in assembled contigs would be a source of direct evidence. The major challenge is that it is often only possible to assemble MGS reads into short contigs, which commonly represent a small fraction of complete genomes and can be enriched for assembly errors. Many existing methods for detecting HGT events in complete genomes cannot be applied to this fragmented data. This challenge is gradually being addressed as deeper sequencing read depths and improvements in related technologies have enabled higher-quality MAGs to be produced, as we discuss in the next sections. Nonetheless, methods specifically focused on poorly assembled MGS data have also been developed. These approaches for identifying putative HGT in both high and low-quality MAGs will be discussed below.

### Composition-Based Approaches

The first major approach developed to detect HGT was based on comparing base and codon usage composition across genes within a genome (Médigue et al. 1991; Lawrence and Ochman 1997). This general approach is motivated by the findings that base composition and codon usage are largely homogenous within a genome (Sueoka 1961; Hildebrand et al. 2010). In addition, base composition is known to be linked to taxonomy: taxa within the same lineage tend to have genomes with similar GC-content and base composition overall (Sueoka 1961). Genomic regions that compositionally differ from background are referred to as “genomic islands.” Two popular tools for identifying these regions are GIST (Hasan et al. 2012) and IslandViewer (Bertelli et al. 2017) although many similar tools are also available (Langille et al. 2010; Lu and Leong 2016).

Composition-based approaches have been applied to MGS contigs (Hemme et al. 2010), but this is not typically performed because longer regions are thought to be required to accurately determine the background composition of the genome. Nonetheless, one composition-based method has been proposed specifically for MGS-assembled contigs (Tamames and Moya 2008). This method involves calculating the frequencies of k-mers within each gene in a contig to generate a vector of frequencies per gene. Pearson correlations between these vectors across genes are then calculated and these genes are clustered by these correlations to enable outlier genes to be identified (fig. 2*B*). The challenge of this method is that the choice of cutoff for distinguishing genes into clusters can have a large effect on the result and it is unclear what value should be used. This approach has been applied previously to fosmid clones corresponding to *Verrucomicrobia* to identify putative HGT events (Kielak et al. 2010).

There are two key limitations of the above compositional approaches. First, false positives can arise simply due to genomic variation in composition, such as variation in base composition related to distance from the replication terminus (Deschavanne and Filipski 1995; Guindon and Perrière 2001). However, this issue is partially addressed in tools that test for differences in k-mer frequencies, which are thought to be more genome-specific (Karlin and Burge 1995), rather than GC-content and codon usage (Koski et al. 2001; Wang 2001). The second major limitation is that ancient HGT events are difficult to detect because transferred genes eventually evolve (or “ameliorate”) to become similar to the rest of the genome (Lawrence and Ochman 1997, 1998). This issue implies that only recently acquired HGT events can be identified using compositional approaches.

### Implicit Phylogenetic Approaches

Another common approach for detecting HGT is to identify genes with higher sequence similarity to homologs encoded by more distantly related taxa compared with close relatives. This class of approach is biased toward more ancient HGT events in contrast to the compositional approaches described above (Ragan et al. 2006). As above, variations on this approach have been implemented for complete genomes (Clarke et al. 2002; Ragan and Charlebois 2002; Podell and Gaasterland 2007). One example is HGTector, which compares protein sequences across genomes of varying phylogenetic distance and includes several improvements that make it resilient to technical and biological confounders (Zhu et al. 2014).

For short MGS-derived contigs, one taxonomic-assignment approach has been proposed that involves running BLASTX (Altschul et al. 1990) on genes within contigs against the GenBank nonredundant database and assigning taxonomy to the genes based on the best-hits (Tamames and Moya 2008). In this case, HGT events are identified when there is sufficient disagreement in the taxonomic assignment of genes within the same contig. WAAFLE (http://huttenhower.sph.harvard.edu/waafle; last accessed August 27, 2019) is a software that implements a similar approach (manuscript in prep). The WAAFLE pipeline involves identifying the most similar matches in a pan-genome database for each gene in a contig. The tool then determines whether the genes in the contig can be explained entirely by a single species or if multiple species are needed to account for the contig gene content. This latter case is taken as putative evidence of HGT. The major strength of this approach is that multiple similarity matches are retained per gene in a contig. This information enables a conservative taxonomic-assignment approach to be employed where contigs can be classified as a single species, even if that species is not the best-hit for each gene, which is intended to reduce the number of false positives.

### Explicit Phylogenetic Approaches

Although the similarity-based HGT-detection methods described above use phylogenetic principles, they do not make direct use of phylogenetic trees to test for phylogenetic incongruencies. Testing for phylogenetic incongruencies refers to comparing a gene tree based on homologous sequences across taxa with the phylogenetic tree for those taxa. The growing number of prokaryotic genomes from pure cultures has enabled large-scale phylogenetic methods to be developed (Beiko et al. 2005; Puigbo et al. 2009). Similar approaches have been applied to MAGs in recent years as well (Guo et al. 2015; Soo et al. 2017). For example, genomes from a basal-branching clade known as Sericytochromatia within Cyanobacteria were assembled from multiple MGS data sets (Soo et al. 2017). These genomes were taxonomically classified based on their 16S ribosomal RNA gene sequences and placement within a supertree of reference genomes was performed based on concatenated protein sequences. These MAGs enabled standard tests for phylogenetic incongruency to be run to identify proteins that were phylogenetically dispersed differently from the supertree, including key genes involved in photosynthesis missing in Sericytochromatia. Despite such successes reconciling gene and species trees remains a nontrivial problem because disagreements in the tree can be biologically due to either gene duplication, HGT, or gene loss. Accordingly, this has been deemed the duplication-transfer-loss reconciliation problem and several methods have been developed to address this issue (Kamneva and Ward 2014). RANGER-DTL is one such method that compares gene and species trees and identifies the most likely positions on the species tree where either speciation, gene duplication, HGT, or gene loss have occurred (Bansal et al. 2018). A similar method is the software GLOOME (Cohen et al. 2010), which is a maximum-likelihood approach that can also be used to infer the position of gene gain and loss events across a phylogenetic tree, but does not consider duplication and speciation events.

### Identifying Putative Gene Transfers in a Defined Community

The methods for detecting HGT presented above approach the problem from a range of perspectives, which can result in strikingly different inferences of HGT. One example is a comparison of a compositional and a sequence similarity approach that resulted in fewer than 5% of HGT events in agreement (Tamames and Moya 2008). This drastic difference and other examples (Ragan et al. 2006) are at least partially due to the differing sensitivity of these tools for detecting HGT events of different ages. Importantly, these tools can be used in combination to yield more robust inferences (Omelchenko et al. 2003; Schoenfeld et al. 2013).

MetaCHIP is a recently published tool that is partially based on this idea, but intended specifically to identify HGT events between observed donor and recipient genomes in a natural community (Song et al. 2019). This tool first performs an all-against-all BLASTN of genes within assembled contigs from a given community. Potential HGT events are identified based on genes with best-hits in other taxonomic groups (e.g., in another family). False inferences due to duplicated regions of contigs are explicitly accounted for in order to reduce the false positive rate. A gene tree is then created for all genes on this short-list of putative HGT events. These trees are compared with a species tree based on 43 universal single-copy genes (USCGs) with the RANGER-DTL software to determine whether HGT, or a different mechanism, better accounts for any phylogenetic incongruencies.

### Characterizing Strain Heterogeneity

Although the majority of HGT-detecting methods are focused on identifying transfers between different species, studying MAGs can also yield insight into population heterogeneity within a species. Because MAGs are based off the genomes of numerous bacterial cells in a community there is always some degree of genetic variation in the reads underlying MAGs. In addition, homologous recombination is known to occur between divergent strains, which can result in mosaic genomes with different gene blocks (Falush et al. 2001; Papke et al. 2004; Dunn et al. 2009). Due to the different assortment of gene blocks between closely related organisms, these recombination events are examples of recent HGT. Leveraging MAGs to assess intraspecies recombination is appealing because recombination hotspots can be readily identified with this datatype.

Key work in this area has focused on identifying large regions of homologous recombination within the genomes of *Leptospirillum* and *Ferroplasma* species originating from acid mine drainage sites (Tyson et al. 2004; Denef et al. 2007; Denef and Banfield 2012). These recombination blocks contain genes hypothesized to be needed for rapid adaptation to this extreme environment. Another recent example was that of a novel archaeon genome assembled from water samples of intermediate and deep aquifers (Probst and Banfield 2018). Not only was the complete genome of the taxon named *Candidatus* “Forterrea multitransposorum” assembled, but population variation of this taxon was also assessed by mapping reads to this assembled genome. Through this approach, the authors identified hotspots of homologous recombination occurring between members of the species. These examples highlight that because MAGs represent a population rather than individual organisms, they can be leveraged to identify regions of recent HGT.

### Leveraging Existing Reference Genomes

Rather than directly identifying HGT in metagenome sequencing data, inferences made from metagenomes have also been used to inform analyses on reference genomes (fig. 2*C*). For instance, putative HGT events in *Synechococcus* reference genomes were identified by mapping MGS reads to these genomes and identifying unmapped regions with divergent trinucleotide composition (Palenik et al. 2009). A different example focused on a peptides/nickel transport complex identified to be enriched in the gut metagenomes of lean individuals (Meehan and Beiko 2012). By placing the sequences of the individual gene families involved in this module into gene trees created from homologous genes in existing reference genomes, it was shown that the phylogenetic position of these genes greatly varied. The only potentially high contributor of all gene families involved in the module was the gut commensal *Faecalibacterium prausnitzii*. Evidence for rampant HGT of this peptides/nickel transport complex was found by focusing on this module within *Faecalibacteriumprausnitzii* reference genomes. More generally, putative HGT events identified in metagenomes can help decrease the search space of gene families and reference genomes to help directly identify individual cases of HGT (Palenik et al. 2009; Meehan and Beiko 2012; Hemme et al. 2016; Llorens-Marès et al. 2017). This approach has proven useful but is highly dependent on existing reference genomes and does not take advantage of the potential to assemble metagenomes.

### Current Barriers and Outlook

There are two main barriers to researchers detecting HGT in MAGs within their own data. The first barrier is the challenge of generating adequate quality MAGs, which is closely linked to the major goal in metagenomics of improving the quality of assemblies overall. This issue can best be addressed through several recent technological advances (see the Potential Avenues to Improve Metagenome Assemblies section). The second obstacle preventing researchers from detecting HGT events is that these analyses require substantial bioinformatics expertise. Determining which approach to use is nontrivial and will largely depend on the biological question. For example, different methods are available if researchers are interested specifically in identifying hotspots of recombination within a single species (Probst and Banfield 2018) or identifying HGT events between different species in a given community (Song et al. 2019). In addition, researchers’ choices can be informed by what time-scale of HGT they are interested in investigating. However, even with a clear research question selecting a specific tool for these analyses can be problematic and so in practice comparing the output of several methods would likely be best. A robust evaluation of the performance of these approaches is needed to better inform researchers’ choice of tools. This evaluation is especially needed for tools applied to MGS-derived contigs and assemblies, which could be done by simulating HGT events within a defined set of genomes with a tool such as HgtSIM (Song et al. 2017). In addition, although existing HGT-detection methods can be applied to high-quality MAGs (fig. 2*D*), this may come at the cost of an unacceptable false positive rate because past evaluations of tool performances have largely been focused on isolate genomes with relatively little contamination.

One reassuring final point is that although different approaches to identify HGT in isolate genomes identify mainly nonoverlapping sets of genes (Ragan et al. 2006), the genes identified tend to be of similar functions (Lawrence and Roth 1996; Beiko et al. 2005; Cordero and Hogeweg 2009; Kanhere and Vingron 2009). Genes related to mobile elements, central intermediary metabolism, amino acid biosynthesis, and energy metabolism are enriched in gene sets identified as horizontally transferred (Jain et al. 1999; Beiko et al. 2005). In contrast, information-processing genes such as ribosomal proteins are less commonly identified as horizontally transferred (Beiko et al. 2005). There are exceptions to this rule, for example, genes related to translation have been found to be commonly horizontally transferred between bacteria, but not between kingdoms (Kanhere and Vingron 2009). These recurrent observations may be related to functions that rely on fewer gene families and regulatory partners being easier to transfer (Lawrence and Roth 1996; Aris-Brosou 2005). In addition, the widespread transfer of metabolism genes is likely related to strong selection for survival in novel environments with limited resources (Lawrence 2001) although the exact genes are environment-specific (Smillie et al. 2011). Identifying putative HGT events enriched for the above functional categories has been previously used as validation that an approach is working (Beiko et al. 2005), which would also be an important output to compare in future evaluations of HGT-detection approaches.

## MAG Quality Control

The major challenge facing the identification of all the genic events described above is poor metagenome assemblies. This issue has recently been commented upon in the context of low-quality MAGs being added into public genome repositories (Shaiber and Eren 2019). Either composite assemblies of multiple taxa or incomplete genomes missing genes of interest could result in incorrect inferences of HGT. One extreme example is of the tardigrade genome, which was falsely identified as having 17% of genes acquired through HGT due to contaminant sequences within the assembly (Koutsovoulos et al. 2016). Such false inferences are more likely in metagenome assemblies compared with genome assemblies due largely to the challenge of distinguishing many organisms at different abundances (Ayling et al. 2019). Misassemblies can also affect the detection of other genic events as well. For instance, repetitive regions of assemblies are difficult to resolve with current short-read sequencing (Chin et al. 2013), which can make duplication events difficult to identify. Due to these challenges, an understanding of the workflows for generating MAGs is needed. Here, we briefly outline the current approaches and issues in metagenome assembly to give the reader a starting point.

There are many metagenome assembly tools currently available, which are predominately based on De Bruijn graphs of overlapping k-mers (Vollmers et al. 2017; Ayling et al. 2019). The outputs of these tools are assembled contigs, which typically vary in length from ∼500 bp to near-complete genomes. Some of the most popular freely available assembly tools are MetaSPAdes (Nurk et al. 2017), Ray Meta (Boisvert et al. 2012), Omega (Haider et al. 2014), IDBA-UD (Peng et al. 2012), and Megahit (Li et al. 2015). Choice of assembly tool can have a major influence on the resulting assembled contigs, and so careful consideration needs to be taken at this stage. An independent evaluation of these and other methods found that MetaSPAdes performed best overall with the caveat that it may not be appropriate for distinguishing highly similar genomes (Vollmers et al. 2017). However, no assembly tool performed best across all environments and it was suggested that the best choice of assembly tool depends on the study environment and research question.

Contig binning, where contigs from the same species or strain are grouped, is another key step when generating MAGs. Binning approaches typically group contigs based on sequence composition (e.g., GC or tetranucleotide content) and similar coverage of mapped reads (Ayling et al. 2019). The most popular freely available binning tools are CONCOCT (Alneberg et al. 2014), MaxBin2 (Wu et al. 2016), and MetaBAT (Kang et al. 2015). As above, the choice of binning software can have drastic effects on the resulting MAGs (Meyer et al. 2018). One partial solution to this issue is to run multiple binning tools and use the software Das Tool (Sieber et al. 2018) to identify the consensus output, which has been shown to produce high-quality bins (Meyer et al. 2018).

Evaluating the quality of MAGs is a crucial step once the final contig bins have been generated and guidelines for how to categorize MAGs based upon quality metrics have recently been established (Bowers et al. 2017). The two key metrics are completeness and contamination, which are based on the counts of USCGs identified in an assembly. Completeness is measured based on the proportion of USCGs identified in an assembly and contamination is defined as the proportion of USCGs found more than once in an assembly. Hard cutoffs for these metrics have been suggested for categorizing the overall quality of a MAG, for instance high-quality draft MAGs are defined as being >90% complete with <5% contamination (Bowers et al. 2017). CheckM (Parks et al. 2015) and BUSCO (Simão et al. 2015) are two tools that will estimate the completeness and contamination of prokaryotic assemblies and BUSCO can also be used to evaluate eukaryotic assemblies. Determining strain heterogeneity, the degree of contamination due to different strains, within an assembly is also important, which can be measured using CheckM or alternatively custom methods to identify polymorphisms in an assembly (Pasolli et al. 2019). An assembly with high strain heterogeneity can still be useful but should be considered differently than an assembly of a single strain.

Importantly, when reporting gene gain and loss events in a set of MAGs it would be important to also report the estimated completeness and contamination within these MAGs. In particular, it would be important to establish within a given MAG that more gene gain and loss events were inferred than are expected given the two quality scores. Ideally, manual validation of inferred gene gain and loss events would also be performed upon assemblies. At minimum this validation would include visually assessing the read coverage across an assembly at the site of the inferred gene gain or loss event. In practice, manually validating many events in this way would not be feasible, but it could be performed for a representative set.

## Potential Avenues to Improve Metagenome Assemblies

**Fig. 3.**
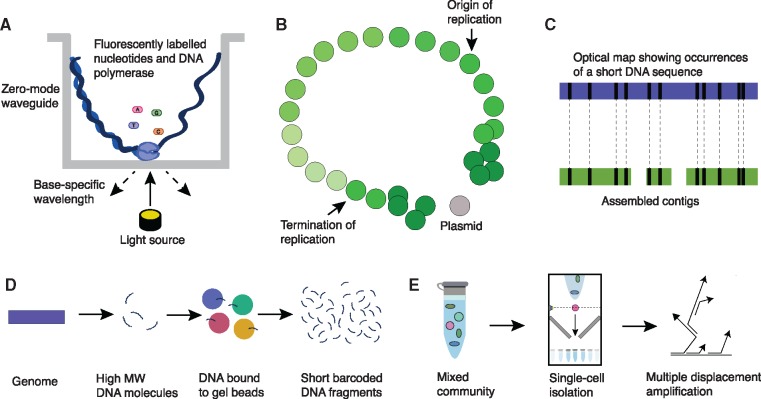
—Promising technologies that could improve metagenome assembly. (*A*) Long-read sequencing as represented by single-molecule real-time (SMRT) sequencing, which takes place in a zero-mode waveguide. Fluorescently labeled nucleotides are added one at a time at the bottom of the well as the new strand of the input DNA is synthesized. The fluorescence of each added nucleotide is measured to determine the sequence. (*B*) Illustration of how relationships between contigs based on chromosome conformation capture can be visualized. This simplified illustration is based on the previously determined relationship between *Escherichia coli* contigs (Marbouty et al. 2014). The darker the shade of green, the higher the contact frequency of contigs. This visualization displays how the contig genomic ordering can be determined through chromosome conformation capture. The contig contact map can be used to improve the scaffolding step of the genome assembly. (*C*) Diagram illustrating principle of optical maps (blue) improving genomic assemblies (green). Solid black bars indicate occurrences of a short DNA sequence along the genome, which can be used to order contigs and correct assembly errors. (*D*) Simplified protocol for barcoding genomic fragments so that reads originating from the same high molecular weight (MW) DNA molecule can be identified. (*E*) Key steps required before single-cell sequencing. Individual cells need to be isolated using one of several technique (e.g., flow cytometry as shown in this panel) and then whole-genome amplification is conducted using multiple displacement amplification. The small arrows indicate amplified regions.

Several recent technologies have been developed which potentially could result in improved MAGs (fig. 3). These technologies include new long-read sequencing approaches, metagenomics chromosome conformation capture, barcoding reads from the same genomic fragment, and optical mapping of short sequences along genomes to inform assembly.

There are two promising long-read technologies currently available: Single-Molecule Real-Time (SMRT; Pacific Biosciences [McCarthy 2010]) and Oxford Nanopore (Mikheyev and Tin 2014) sequencing. SMRT sequencing involves binding a custom DNA polymerase with bound DNA to be sequenced at the bottom of a zero-mode waveguide (Slatko et al. 2018) (fig. 3*A*). Fluorescently labeled nucleotides are added to the growing chain, which enables each base to be identified as it is added. The key advantage of this approach is that long reads typically in the range of 10–15 kb (but ranging up to 50 kb or larger) can be produced (Slatko et al. 2018). SMRT sequencing reads generally contain 11–14% incorrect bases, but consensus sequences between overlapping sequences can be used for correction because the errors are random (Roberts et al. 2013). In contrast, short-read sequencing approaches result in nonrandom errors, which are more difficult to correct. This approach has been used mainly with hybrid approaches with short-read sequencing to make improved assemblies of isolate genomes (Koren et al. 2012). However, algorithms have been developed to error-correct the reads so that they can be used alone for high-quality assembly (Chin et al. 2013). Importantly, these long reads are better able to sequence regions that are problematic with short-read approaches, including repetitive regions. New SMRT sequencing approaches are being developed, including circular consensus sequencing, which provides higher accuracy through repeated sequencing of the same circularized fragment of 500–2,500 nucleotides in length. This approach has been shown to result in improved assembled contigs compared with Illumina HiSeq sequencing on the same samples (Frank et al. 2016).

Nanopore sequencing refers to passing a strand of DNA through a nanopore and measuring the changing current, which differs depending on the bases passing through. This method also results in extremely long reads, typically in the range of 13–20 kb, and is rapidly improving in throughput (Tyson et al. 2018). The main down-side of this approach is the high error rate, which can range up to 40% of bases being incorrect (Laver et al. 2015). Nonetheless, this technology has been used to successfully assemble the *E. coli* K-12 mG1655 genome at 99.5% base accuracy by first making multiple-sequence alignments of nanopore reads to correct read errors (Loman et al. 2015). This technology is especially useful for resolving large repetitive regions and merging contigs derived from short-read data, as was recently demonstrated through improvements to the *Caenorhabditis elegans* reference genome (Tyson et al. 2018).

These evaluations of long-read sequencing technology are promising and both technologies will likely confer similar improvements to MAGs in the future. At least one example of improved quality of the continuity of MAGs has been demonstrated based on nanopore sequencing of a complex bioreactor community (Arumugam et al. 2019). We expect that many more of these examples will be published as long-read sequencing becomes more widely available. However, despite this promising example, MGS data present many novel challenges and will take additional work to integrate into most bioinformatics pipelines for processing long-read data. For instance, both correcting read errors and resolving repetitive regions with nanopore sequencing would be considerably more challenging with MGS data due to the added complication of strain variation and homologous DNA between different species within the same community. MGS-specific software for processing long-read data is beginning to become available (Kolmogorov et al. 2019), but clear best practices remain to be determined.

Alternative approaches that could improve MAG quality are based on binning genomes prior to or independent of sequencing. One exciting development is Hi-C sequencing, which is an extension of chromosome conformation capture sequencing (Belton et al. 2012). This approach involves crosslinking DNA with formaldehyde, followed by digestion, and then religation so that interacting DNA fragments are ligated together. The novel addition in Hi-C is that a biotin-labeled nucleotide is incorporated at ligation junctions, which makes it much easier to purify out chimeric ligations and identify 3D interactions. This approach has mainly been used to identify long-range interactions, such as between enhancers and promoters (Ron et al. 2017) and to map genome conformational dynamics (Lieberman-Aiden et al. 2009). However, this method can also be exploited to improve genome assemblies by building probability maps of genetic interactions (Burton et al. 2013) (fig. 3*B*), based on the observation that intrachromosomal interactions are much more common than interchromosomal interactions, even at long distances (Lieberman-Aiden et al. 2009). As a proof-of-concept, this approach has been used to distinguish genomes within mock communities and contributed to the assembly of high-quality MAGs of bacterial, archaeal, and fungal genomes (Burton et al. 2014). In addition, Hi-C sequencing has been used to supplement MGS assembly of natural communities in river sediment (Marbouty et al. 2014) and cow rumen samples (Stewart et al. 2018).

Optical mapping is another approach that has been shown to improve genome assembly quality (Hastie et al. 2013). The most recent implementation of this approach is provided by the company BioNano (https://bionanogenomics.com; last accessed August 27, 2019). Their method involves annealing fluorescent probes that bind specific short sequences in DNA. The DNA is then passed through narrow chambers that require a DNA molecule to be passed through in a straight line. The location of the fluorescent probes along genomic fragments is captured as DNA passes through the chamber. This approach has recently been applied to human samples to identify SVs and translocation events (Mak et al. 2016). Optical mapping data could potentially be integrated with MGS data to improve MAG qualities by assigning contigs to the same genome and to order and rearrange contigs within a genome (fig. 3*C*). However, this approach has not been applied to samples from natural communities and it remains unclear what challenges would be faced. A proof-of-concept of this technology applied to mock and natural communities is required for this approach to be evaluated properly.

Another promising approach involves barcoding reads from the same genome prior to MGS (fig. 3*D*). This technology, developed by 10x Genomics (https://www.10xgenomics.com; last accessed August 27, 2019), results in sets of reads that are derived from the same genomic fragment. This information is especially useful for resolving SVs and repeats and for phasing variants (Bishara et al. 2015; Zheng et al. 2016). These “read clouds” could be leveraged in an analogous way to Hi-C sequencing data to produce improved MAGs. It was recently shown that this datatype can be used to generate high-quality MAGs from mock and natural communities with a barcode-aware assembler called Athena (Bishara et al. 2018). Further published work confirming this finding is required, but this promising result highlights that leveraging “read clouds” could be a straight-forward method for improving MAG qualities.

Lastly, single-cell metagenomics has recently been suggested as an improved approach for isolating individual genomes from mixed communities (Xu and Zhao 2018). This technique involves first isolating individual cells in a sample, extracting the DNA, and performing whole-genome amplification before conducting library preparation and sequencing (fig. 3*E*). The whole-genome amplification step can result in a high proportion of chimeric reads (Lasken and Stockwell 2007) and uneven read coverage (Xu and Zhao 2018), which can complicate genome assembly. Nonetheless, single-cell metagenomics has been performed successfully, especially when identifying phage and the corresponding bacterial host genomes (Labonté et al. 2015; Munson-Mcgee et al. 2018). In addition, single-cell metagenomics has been integrated with MGS on numerous occasions to improve MAGs (Dupont et al. 2012; Dong et al. 2016; Ji et al. 2017; Yu et al. 2017). Many questions remain regarding the best practices of single-cell metagenomics and the feasibility at high throughput (Xu and Zhao 2018), but this technology is an extremely promising approach to improve MAG quality.

## Conclusions

Several bioinformatics approaches have been applied for identifying HGT events specifically in MGS data, but these approaches have not been extensively benchmarked and there are no clear best practices. These approaches have also been largely custom bioinformatics pipelines that are difficult to compare across studies, but there are several recently developed methods like WAAFLE and MetaCHIP that are now available as stand-alone tools. In addition, currently there are limited bioinformatics approaches to identify gene loss, de novo genes, and gene duplications in MGS data sets. Although inferring these events will become easier as the quality of MAGs improves, there is still a need to develop methods to detect these events in poorly assembled MGS data sets. These methods are needed to better analyze existing MGS data sets and also to study communities with high richness (e.g., soil samples), for which it will likely remain unfeasible to sequence at sufficient coverage to produce many high-quality MAGs in the near future.

There are also many open questions about how to scan for HGT in assembled genomes. Currently, genomic context and potential transfer mechanisms are not directly integrated into HGT-detection pipelines. Automatically identifying corroborating evidence for a transfer, such as the presence of nearby prophage sequences, could help identify recent HGT events, which has been previously argued (Zaneveld et al. 2008). The feasibility of such an approach and whether it would improve HGT event identification accuracy is unclear. Similarly, taxonomic-specific features such as DNA-uptake sequences in DNA imported through transformation (Davidsen et al. 2004) could directly be used to inform inferences.

Regardless of which bioinformatics approach is used, inferences of gene gain and loss in MGS data sets will continue to improve as higher-quality MAGs are produced. The promising technologies outlined above are still in the early stages of application and there remain many open questions. For instance, whether optical mapping can be accurately applied to mixed communities remains unclear. Additional validation of this approach in conjunction with MGS data is necessary to determine whether it would actually improve MGS assemblies. In addition, there are several limitations specific to individual technologies, such as long-read sequencing and Hi-C requiring larger biomass samples. Benchmarking of all of these promising methods is required on natural communities of varying richness, complexity, and biomass to evaluate whether these methods should be differentially applied depending on sample-type or whether general best practices could be developed using only a subset of approaches.
